# The generation mechanism of spike-and-slow wave discharges appearing on thalamic relay nuclei

**DOI:** 10.1038/s41598-018-23280-y

**Published:** 2018-03-21

**Authors:** Bing Hu, Yu Guo, Feng Shi, Xiaoqiang Zou, Jing Dong, Long Pan, Min Yu, Chaowei Zhou, Zhang Cheng, Wanyue Tang, Haochen Sun, Luonan Chen

**Affiliations:** 1 Institute of Applied Mathematics, Department of Mathematics and Statistics, College of Science, Huazhong Agricultural University, Wuhan, 430070 China; 2 Key Laboratory of Systems Biology, CAS center for Excellence in Molecular Cell Science, Innovation Center for Cell Signaling Network, Institute of Biochemistry and Cell Biology, Shanghai Institute of Biological Sciences, Chinese Academy of Sciences, Shanghai, 200031 China

## Abstract

In this paper, we use a model modified from classic corticothalamic network(CT) to explore the mechanism of absence seizures appearing on specific relay nuclei (SRN) of the thalamus. It is found that typical seizure states appear on SRN through tuning several critical connection strengths in the model. In view of previous experimental and theoretical works which were mainly on epilepsy seizure phenomena appearing on excitatory pyramidal neurons (EPN) of the cortex, this is a novel model to consider the seizure observed on thalamus. In particular, the onset mechanism is different from previous theoretical studies. Inspired by some previous clinical and experimental studies, we employ the external stimuli voltage on EPN and SRN in the network, and observe that the seizure can be well inhibited by tuning the stimulus intensity appropriately. We further explore the effect of the signal transmission delays on seizures, and found that the polyspike phenomenon appears only when the delay is sufficiently large. The experimental data also confirmed our model. Since there is a complex network in the brain and all organizations are interacting closely with each other, the results obtained in this paper provide not only biological insights into the regulatory mechanisms but also a reference for the prevention and treatment of epilepsy in future.

## Introduction

Absence epilepsy, a kind of primary generalised seizure diseases, has a strong relationship with the pathological discharge rhythms of bilateral corticothalamic network^1^, and closely associated with action abort, loss of awareness, muscle rigidity, and behavioural arrest, etc.^2,3^. Clinically, it is usually characterized by a brief interference in consciousness, along with typical synchronous and bilateral spike-and-wave discharges (SWDs) with slow frequency (approximately 2–4 Hz) on electroencephalography (EEG) of epilepsy patients^4,5^. This disorder primarily affects children and young adolescents. Although there have been different viewpoints on the origin regions of absence seizures, the ex act initial positions are still unknown. Many results have supported the opinion that seizures arise from abnormal interactions among the specific relay nuclei (SRN) of the thalamus, the thalamic reticular nucleus (TRN) and the cerebral cortex structure^6–8^, which constitute the corticothalamic network (CT). A variety of experimental evidence is presented in Section 8.1 of^9^. Additionally, the classical clinical electroencephalogram (EEG) waveform often appears in the corticothalamic circuit, especially in the cortex^10,11^. Many theoretical studies have investigated the mechanism of epilepsy onset in the cortex structure, particularly, by using the mean field model, and the results agree well with the relative experiments^12–20^.

The thalamus is composed of the TRN and SRN, which play an important role in the production of seizures^21,22^. The typical epileptic seizure EEG waveform has also been observed on the thalamus in some clinical medical experiment^23,24^. In addition, many experiments support the theory that disrupting aminobutyric acid (GABAergic) inhibition role within the thalamus may be a critical factor to produce abnormal oscillations during seizures^25–27^. For instance, increased thalamic tonic GABA inhibition has recently been observed in a model^28^ and in human childhood absence epilepsy^29^. Seo observed that there are region-specific changes in both phasic and tonic *GABA*_*A*_R subunit expression in the thalamus of an epileptic model, which occur prior to seizure onset^30^. Therefore, the thalamus organization has gradually been selected as the target region for deep brain stimulation (DBS), and the control effect was remarkable for some intractable seizures^31–34^. For example, Klinger *et al*. reviewed the patient outcomes and adverse events related to DBS with various stimulation targets and determined that the DBS may be a safe and effective treatment option for refractory epilepsy^35^. However, there are few theoretical studies that evaluate the generation mechanism of seizures originating from the thalamus directly. Therefore, the underlying physiological processes of these seizures are still unclear, leading to limited understanding of the effect caused by various onset mechanisms.

Another interesting question is whether or not the seizure obtained in the theoretical model can be controlled. As is well known, the brain has a complex network where all organizations closely interact with each other. For instance, the oscillating activity of the basal ganglia (including the substantia nigra pars reticulata (SNr), striatal neurons, globus pallidus tissue and subthalamic nucleus) may exert a great effect on physiological states appearing in CT, especially in epilepsy seizure activity^36,37^. Recently, Chen *et al*. reported that absence seizures observed on excitatory pyramidal neurons (EPNs) may be well controlled by tuning the activation level of the SNr^37^ and by changing the coupling weigh of the direct pallido-cortical pathway^38,39^. Hu *et al*. explored the generation mechanism of absence epilepsy appearing on EPN, which is caused by some excitatory pathways projected to the SRN, and they observed that these seizures can be well controlled by appropriately tuning the output from the basal ganglia to the thalamus^40^. Additionally, Hu *et al*. used an external DBS voltage on the EPN, striatum, SNr and thalamus in a basal ganglia-corticothalamic mean field network and reported that seizures appearing on EPN may be inhibited through changing the period and duration of the current into suitable ranges^40–42^. However, the controlling problem on seizures in the thalamus has not been involved by now.

Inspired by the previous study, the current study is based on a model modified from the classic CT network^12–20,37–43^. We first evaluate the onset mechanism of seizures appearing on the SRN and then explore the control mechanism by employing external stimuli on the EPN and SRN. We observe that the typical absence seizure may appear on the SRN upon changing several critical coupling strengths and delays in the model. The polyspike wave can only be induced when the delay is sufficiently large. All of seizures can be controlled by tuning the strength of external stimulus appropriately. Through careful analysis, we also find that the TRN could not show obvious attack phenomena based on our model, and hence the discussion on TRN was omitted in the paper. Due to the complicated internal environment and anatomical structure of the brain, our results may provide not only biological insights into the regulatory mechanisms but also a theoretical reference for the prevention and treatment of epilepsy.

## Model description and numerical calculation method

**Figure 1:**
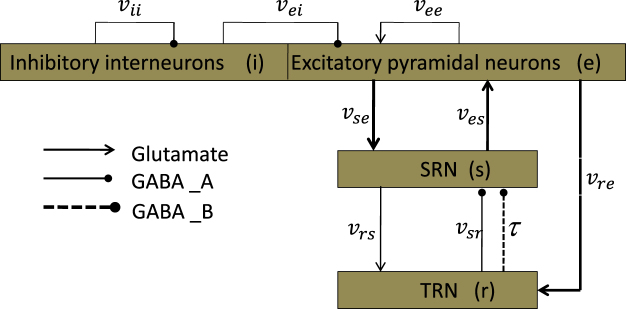
The framework of the CT network. The neural masses are abbreviated as follows: r = reticular nuclei; s = specific relay; i = inhibitory cortical; e = excitatory cortical. Each box represents a nerve nuclei. Arrow heads represent excitatory inputs mediated by glutamate; round heads represent inhibitory connections regulated by the *GABA*_*A*_ (solid lines) and *GABA*_*B*_ (dashed lines) receptors.

Many previous studies have demonstrated the predictive and descriptive validity of the mean-field approach for modelling a wide range of healthy states as well as generalized seizures in human^12–20,37–43^. In this paper, we also use the mean-field equation to describe the corticothalamic network. Here, we provide the schematic diagram of the model used in the paper in Fig. 1, which is modified from the classic CT network with four neural populations^12–20,37–43^. For simplicity, these populations are denoted as follows: s = SRN; r = TRN; i = inhibitory interneurons (IIN); e = EPN. We employ different types of lines to represent various projections between populations. Arrow heads represent excitatory inputs mediated by glutamate; round heads represent inhibitory connections regulated by the *GABA*_*A*_ (solid lines) and *GABA*_*B*_ (dashed lines) receptors. We first study the generation mechanism in the corticothalamic network and then evaluate the controlling mechanism by using external voltage stimuli on the EPN and SRN. Finally, the effect of the delay on seizures is discussed.

Now, we derive equations to describe the dynamic characteristics of nerve nuclei.

The mean firing rate *Q*_*a*_ of “a” (*a* = *s*, *r*, *i*, *e*) is a sigmoid function of the cell-body potential *V*_*a*_,

1 $${Q}_{a}(t)\equiv F[{V}_{a}(t)]=\frac{{Q}_{a}^{max}}{1+exp[\,-\,\frac{\pi }{\sqrt{3}}\frac{{V}_{a}(t)-{\theta }_{a}}{\sigma }]}$$

${Q}_{a}^{max}$ represents the maximum firing rate of “a”, *θ*_*a*_ represents the mean threshold potential, and *σ* is the standard deviation of firing thresholds. $\frac{\pi }{\sqrt{3}}$ is a normalizing parameter obtained by fitting Formula (1) to the error function^15^. It is indicated that the population “a” will discharge at the rate *Q*_*a*_ when the *V*_*a*_ exceeds the *θ*_*a*_. The sigmoid function can ensure that the firing rate of “a” does not exceed ${Q}_{a}^{max}$. It is in line with biological significance, because real neurons cannot fire infinitely quickly.

The variation of the cell-body potential *V*_*a*_ is described by the following differential equations,

2 $${D}_{\alpha \beta }{V}_{a}(t)={{\rm{\Sigma }}}_{b\in B}S({v}_{ab}\mathrm{)}.{v}_{ab}.{\varphi }_{b}(t)$$

3 $${D}_{\alpha \beta }=\frac{1}{\alpha \beta }[\frac{{\partial }^{2}}{\partial {t}^{2}}+(\alpha +\beta )\frac{\partial }{\partial t}+\alpha \beta ]$$

*D*_*αβ*_ is a differential operator representing dendritic and axon integrals of pulse signals. *α* and *β* represent the decay and rise rates of *V*_*a*_. *v*_*ab*_ is the coupling weight in the pathway “*b* → *a*”. *B* represents a set of all neural masses projected to “a”. S(*ν*_*ab*_) = 1 means the projection in the pathway “*b* → *a*” is excitatory; otherwise, we let S(*ν*_*ab*_) = −1. *ϕ*_*b*_(*t*) represents the output pulse rate of the mass “b”. Below, we will introduce a delay *τ* to describe the slow synaptic kinetics on the pathway “*TRN* → *SRN*”, which is induced by the second messenger process.

We employ the following damped wave equation to depict the propagation effect of the axonal field *ϕ*_*a*_,

4 $$\frac{1}{{\gamma }_{a}^{2}}[\frac{{\partial }^{2}}{\partial {t}^{2}}+2{\gamma }_{a}\frac{\partial }{\partial t}+{\gamma }_{a}^{2}]{\varphi }_{a}(t)={Q}_{a}(t)$$

where *γ*_*a*_ = *v*_*a*_/*r*_*a*_ is the damping rate of the population, *v*_*a*_ represents the velocity of pulse conduction, and *r*_*a*_ represents the characteristic axonal range. Inhibitory neurons have only short range projections, compared to excitatory neurons^12,44^. Therefore, we suppose that the axons of IIN and TRN are both too short to support pulse conduction; it is suggested that *ϕ*_*d*_ = *F*(*V*_*d*_)(*d* = *i*, *r*). The pulse propagation effects of EPN and SRN are represented as follows,

5 $$\frac{1}{{\gamma }_{e}^{2}}[\frac{{\partial }^{2}}{\partial {t}^{2}}+2{\gamma }_{e}\frac{\partial }{\partial t}+{\gamma }_{e}^{2}]{\varphi }_{e}(t)={Q}_{e}(t)$$

6 $$\frac{1}{{\gamma }_{s}^{2}}[\frac{{\partial }^{2}}{\partial {t}^{2}}+2{\gamma }_{s}\frac{\partial }{\partial t}+{\gamma }_{s}^{2}]{\varphi }_{s}(t)={Q}_{s}(t)$$

Because such a large system of DDEs is still computationally expensive, it is naturally desirable to make further simplifications. As suggested by the anatomy of the brain, cortical inhibitory interneurons are relatively sparse compared to pyramidal cells (a ratio of 1:4 in favour of inhibitory interneurons). Therefore, we set *Q*_*i*_ = *Q*_*e*_ and *V*_*i*_ = *V*_*e*_ in our model, as similarly treated in many previous studies^12,20,37,40,41^.

Finally, we rewrote equations (1)–(6) in the first-order form, as delay differential equations, to describe the model in Fig. 1. By implementing this reduction, our model clearly becomes computationally more tractable.

7 $$\frac{d{\varphi }_{e}(t)}{dt}={\dot{\varphi }}_{e}(t)$$

8 $$\frac{d{\dot{\varphi }}_{e}(t)}{dt}={\gamma }_{e}^{2}[-{\varphi }_{e}(t)+F({V}_{e}(t))]-2{\gamma }_{e}{\dot{\varphi }}_{e}(t)$$

9 $$\frac{d{\varphi }_{s}(t)}{dt}={\dot{\varphi }}_{s}(t)$$

10 $$\frac{d{\dot{\varphi }}_{s}(t)}{dt}={\gamma }_{s}^{2}[-{\varphi }_{s}(t)+F({V}_{s}(t))]-2{\gamma }_{s}{\dot{\varphi }}_{s}(t)$$

11 $$\frac{dX(t)}{dt}=\dot{X}(t),\,X(t)={[{V}_{e}(t),{V}_{r}(t),{V}_{s}(t)]}^{T}$$

12 $$\frac{d{\dot{V}}_{e}(t)}{dt}=\alpha \beta ({v}_{ee}{\varphi }_{e}+{v}_{ei}F({V}_{e})+{v}_{es}{\varphi }_{s}(t)-{V}_{e}(t))-(\alpha +\beta ){\dot{V}}_{e}(t)$$

13 $$\frac{d{\dot{V}}_{r}(t)}{dt}=\alpha \beta ({v}_{re}{\varphi }_{e}+{v}_{rs}{\varphi }_{s}(t)-{V}_{r}(t))-(\alpha +\beta ){\dot{V}}_{r}(t)$$

14 $$\frac{d{\dot{V}}_{s}(t)}{dt}=\alpha \beta ({v}_{se}{\varphi }_{e}+{v}_{sr}^{A}F({V}_{r})+{v}_{sr}^{B}F({V}_{r}(t-\tau ))-{V}_{s}(t))-(\alpha +\beta ){\dot{V}}_{s}(t)$$

Calculation methods of previous studies involving the mean field model of the corticothalamic system were mainly based on the numerical continuation-tools, such as the matlab package DDE-BIFTOOL and PDDE-CONT^20,43^. In contrast to the study in^20,43^, all simulations in this paper are performed in the Matlab environment by employing the standard fourth-order Runge-Kutta method, with a integral step of Δ*t* = 0.00005 s. First, we performed the bifurcation analysis for several critical coupling strengths to explore the transition mechanism between four states. The bifurcation parameter diagram was obtained by taking the local maximum and minimum values of *ϕ*_*s*_ through changing one parameter. All numerical calculations are performed for a sufficient time, and the values taken from the stable time series are adopted. Similarly, we can easily obtain the two-dimensional state bifurcation diagram by adopting the above analysis technology. By using the fast Fourier transform on the time series of *ϕ*_*s*_, we can obtain the power spectral density of the SRN. Generally, the spike-wave discharge frequency is the highest at the beginning of the burst and decreases through the seizure, as observed in the experiment. Therefore, we taken the maximum peak frequency as the oscillation dominant frequency in this paper. Unless otherwise noted, all parameter values employed in numerical calculation are listed in the appendix. The external stimuli are small voltages applied on the SRN and EPN directly.

## The generation mechanism of seizures by changing several critical coupling strengths

**Figure 2:**
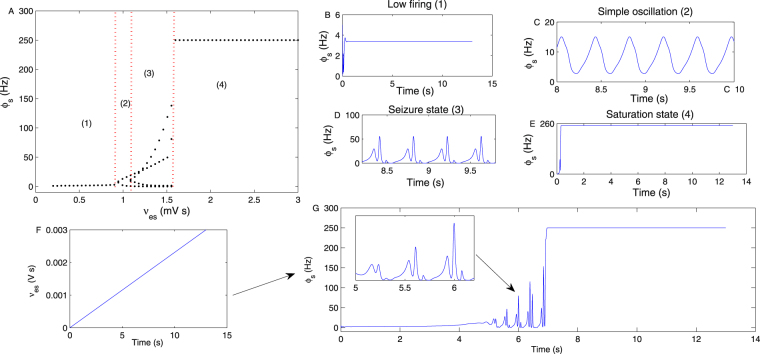
(**A**) The transition process of *ϕ*_*s*_ by changing the coupling strength *v*_*es*_. (1), (2), (3) and (4) represent the low firing state, the simple oscillation state, the seizure state and the saturation state, respectively. (**B**–**E**) Four specific time sequences of *ϕ*_*s*_, simulated by setting *ν*_*es*_ = 0.6 mV s, *ν*_*es*_ = 1.05 mV s, *ν*_*es*_ = 1.4 mV s and *ν*_*es*_ = 1.8 mV s, respectively. (**F**) The linear increase in *v*_*es*_. (**G**) The time series bifurcation diagram of *ϕ*_*s*_ with a linear increase in *v*_*es*_.

Figure 2A is the bifurcation diagram of *ϕ*_*s*_ obtained by varying the coupling strength *v*_*es*_. With an increase in *v*_*es*_, four states (1)–(4) appear. With each fixed *v*_*es*_, a different oscillatory frequency and number of points in the diagram represent four different states. Figure 2B–E are four specific time sequences of *ϕ*_*s*_, obtained by setting *ν*_*es*_ = 0.6 mV s, *ν*_*es*_ = 1.05 mV s, *ν*_*es*_ = 1.4 mV s and *ν*_*es*_ = 1.8 mV s in Fig. 2A, respectively. State (1) is the low firing state with a relative low discharge frequency, which usually represents the normal state of the brain. State (2) is the simple oscillation state, consisting of periodic sinusoidal-like activity; it is a transition state between the normal state and the epileptic seizure state in the brain, which is often observed on the EEG of patients. State (3) indicates the typical epileptic seizure state, which represents a type of periodic activity, with four extreme points of oscillation frequency in one period. State (4) represents the saturation state with an oscillation frequency of 250 Hz (i.e., ${Q}_{s}^{max}$). When *v*_*es*_ increases to approximately 0.9 mV s, the low firing state is transferred to the simple oscillation state by a Hopf bifurcation. With a further increase in *v*_*es*_, to approximately 1.1 mV s, the simple oscillation state is transferred to the typical epileptic seizure state. This mechanism is often defined as the spike bifurcation, induced by an inflection point of the vector field^13,20^. Finally, if *v*_*es*_ is too large, such as beyond 1.6 mV s in Fig. 2A, the SRN enters into a saturation state. The mechanism of these transitions may be easily explained in Fig. 1. With an increase in *v*_*es*_, the firing level of the EPN is enhanced, which will lead to increased excitability output from the EPN to the SRN. Moreover, the firing activation level of SRN is enhanced with an increase in *v*_*es*_. Therefore, the SRN shows states (1), (2), (3) and (4) in order, as shown in Fig. 2A. Figure 2F exhibits the linear increase in *v*_*es*_ with time. Figure 2G is the time series bifurcation diagram of *ϕ*_*s*_ with linearly increasing *v*_*es*_, which compares very well with Fig. 2A.

**Figure 3:**
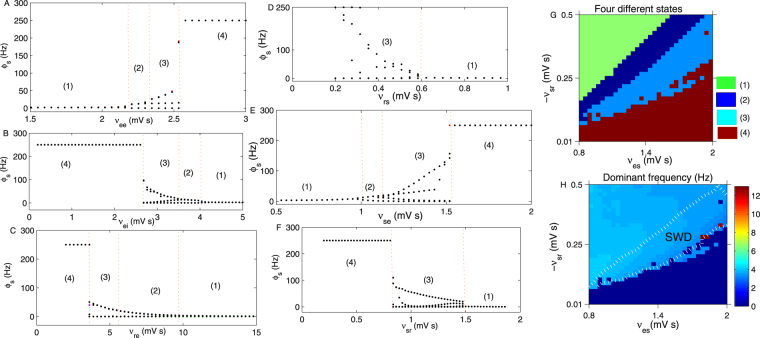
(**A**) The bifurcation diagram of *ϕ*_*s*_ obtained by varying the coupling strength *v*_*ee*_. We set *τ* = 1.8 s, −*v*_*ei*_ = 3.6 mV s and *v*_*se*_ = 1.4 mV s. (**B**) The bifurcation diagram of *ϕ*_*s*_ obtained by varying the coupling strength *v*_*ei*_. Here, we set *τ* = 0.7 s and *v*_*se*_ = 2.4 mV s. (**C**) The bifurcation diagram of *ϕ*_*s*_ obtained by changing the coupling strength *v*_*re*_. We set *τ* = 2.7 s and *v*_*se*_ = 2.4 mV s in (**C**). (**D**) The bifurcation diagram of *ϕ*_*s*_ obtained by varying the coupling strength *v*_*rs*_. We set −*v*_*sr*_ = 1.2 mV s. (**E**) The bifurcation transition of *ϕ*_*s*_ obtained by tuning *v*_*se*_. (**F**) The bifurcation process of *ϕ*_*s*_ with increasing in −*v*_*sr*_. We set *τ* = 0.06 s and *v*_*se*_ = 2.4 mV s in (**F**). (**G**) The state transition diagram in (*ν*_*es*_, −*ν*_*sr*_). By varying the parameter values, four states (1)–(4) appeared. (**H**) Dominant frequency calculation results in the panel (*ν*_*es*_, −*ν*_*sr*_). The 2–4 Hz epileptic seizure region is marked as “*SWD*”, which is surrounded by the white dotted lines. In all simulations of (**G**,**H**), we set *ν*_*rs*_ = 1.2 mV s.

Similarly, we next evaluate the effect of several other critical coupling strengths on the oscillation state of the SRN. Figure 3A presents the bifurcation diagram of *ϕ*_*s*_ obtained by varying the coupling strength *v*_*ee*_. The enhanced strength of *v*_*ee*_ can increase the activation level of the EPN, which in turn improves the firing level of the SRN. Therefore, states (1), (2), (3) and (4) appear in order with an increase in *v*_*ee*_. Figure 3B presents the bifurcation diagram of *ϕ*_*s*_ obtained by varying the coupling strength *v*_*ei*_. *v*_*ei*_ is the inhibitory coupling strength from the IIN to the EPN; thus, increasing *v*_*ei*_ can decrease the firing level of the EPN, which in turn reduces the activation level of the SRN. Therefore, the saturated state (4) only appears when the *v*_*ei*_ is relative small. When the *v*_*ei*_ increases sufficiently (approximately −4 mV s), the SRN enters into the low firing state. Therefore, states (4), (3), (2) and (1) appear successively in Fig. 3B. The increase in *v*_*re*_ can enhance the activation level of TRN, which strengthens the inhibitory output from the TRN to the SRN, and hence, the firing activity of the SRN is reduced in this case. Therefore, the qualitative bifurcation diagram of *ϕ*_*s*_ obtained by varying *v*_*re*_ is similar to Fig. 3B, as shown in Fig. 3C. Figure 3D is the bifurcation diagram of *ϕ*_*s*_ obtained by changing *v*_*rs*_, which is the excitatory coupling strength from the SRN to the TRN. The increase in *v*_*rs*_ can enhance the activation level of the TRN, which leads to enhanced inhibition from the TRN to the SRN. Therefore, the seizure may appear only when the *v*_*rs*_ is relatively small. When the *v*_*rs*_ is sufficiently large, approximately to 0.6 mV s, the SRN is pushed into the low firing state. Because the *v*_*se*_ is the direct excitatory coupling strength from the EPN to the SRN, similar to Figs 2A and 3A, the four states (1), (2), (3) and (4) appear in sequence with an increase in *v*_*se*_, as shown in Fig. 3E. −*v*_*sr*_ is the direct inhibitory coupling strength from the TRN to the SRN. Therefore, increasing the −*v*_*sr*_ can reduce the activation level of the SRN. As shown in Fig. 3F, the saturated state only appears when −*v*_*sr*_ is relatively small; when the −*v*_*sr*_ increases to approximately 0.85 mV s, the seizure state appears. If the inhibition is too large (approximately 1.5 mV s), SRN shocking occurs with a low constant frequency. By combining two arbitrary coupling strengths from the above single parameter bifurcation diagram, we can obtain the bifurcation diagram in the two-dimensional plane. For example, Fig. 3G is the state bifurcation analysis obtained in (*ν*_*es*_, −*ν*_*sr*_). By varying the parameter values, states (1)–(4) appeared, denoted by different colours. Figure 3H is the corresponding dominant frequency analysis in the panel (*ν*_*es*_, −*ν*_*sr*_). We find that the epileptic seizure frequency falls approximately in 2–4 Hz, denoted as “SWD” in Fig. 3H.

## Inhibit of seizures by using the external stimulation

An additional question is whether or not the epileptic seizure observed on the SRN can be controlled in our model. As is well known, the seizure is primarily induced by abnormal interactions between the cortex and the thalamus, i.e., the corticothalamic system. Therefore, the epileptic seizure may be regulated by other brain tissues that are closely related to the corticothalamic system, such as the basal ganglia. Considering the anatomical structure of the brain, the globus pallidus externa (GPe) may project the direct GABAergic inhibitory connection to the cortex^38,39^, the SNr demonstrates inhibitory projections to the thalamus by the direct pathway^37–42^ and the indirect pathway^45^, and the subthalamic nucleus (STN) usually has a constant nonspecific input onto the SRN^37–42^. The typical absence seizures appearing on the cortex can be well controlled by these connections to the corticothalamic system^37–42^. Inspired by the above studies, in this section, we employ some external stimulations on the EPN, TRN and SRN, to explore whether or not the seizures observed in our model can be inhibited and to determine its controlling mechanism.

### Regulation of seizures by the external stimulation acting on the SRN

**Figure 4:**
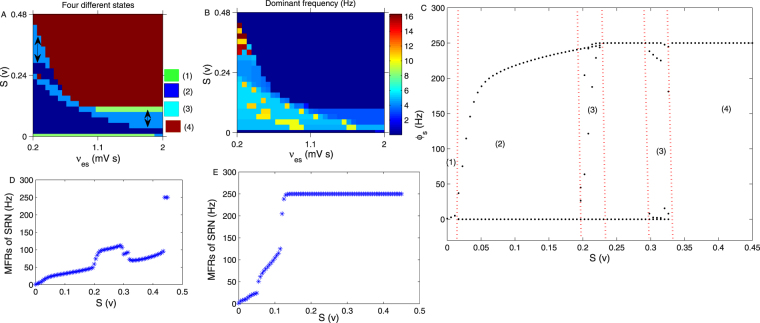
(**A**,**B**) The state and dominant frequency bifurcation results in (*ν*_*es*_, *S*). S represents the external stimuli applied on the SRN. The seizure may be inhibited by both increasing and decreasing the strength of S, implied by bidirectional arrows in (**A**). The dominant frequency of seizures falls approximately in the range of 2–4 Hz, easily found in (**B**). Here, we set −*ν*_*sr*_ = 1.2 mV s. (**C**) A typical transition process of *ϕ*_*s*_ obtained by setting *ν*_*es*_ = 0.4 mV s in (**A**). (**D**,**E**) The MFRs of the SRN, obtained by setting *ν*_*es*_ = 0.4 mV s and *ν*_*es*_ = 1.5 mV s in Fig. 4A, respectively. They show that the changing trend of MFRs is consistent with that in the bifurcation diagram in Fig. 4A.

Figure 4A,B are the state and dominant frequency analysis in (*ν*_*es*_, *S*), respectively. S represents the external stimulus voltage applied on the SRN. It is shown that the seizure may be inhibited by both increasing and decreasing the strength of S, as implied by the bidirectional arrows in Fig. 4A. This is an obvious bidirectional control phenomenon. The dominant frequency of the state (3) almost falls in 2–4 Hz, as shown in Fig. 4B. Figure 4C is a typical bifurcation process of *ϕ*_*s*_ by setting *ν*_*es*_ = 0.4 mV s in Fig. 4A. Here, we can more clearly see the state transition process among the four states. To better understand the control mechanism, we simulated the mean firing rates (MFRs)^37^ of the SRN by increasing S. Figure 4D,E were obtained by setting *ν*_*es*_ = 0.4 mV s and *ν*_*es*_ = 1.5 mV s in Fig. 4A, respectively. The figures show that the trend of MFRs is consistent with that in the bifurcation diagram.

**Figure 5:**
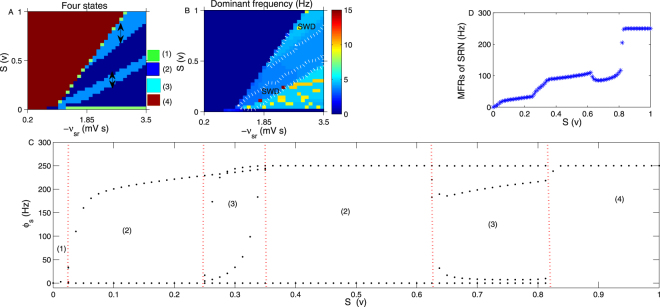
(**A**,**B**) The state and dominant frequency bifurcation diagram in the two-dimensional panel (−*ν*_*sr*_, *S*). S is the external voltage employed on the SRN. The state (3) may be inhibited via either decreasing or increasing S, as marked by bidirectional arrows in (**A**). The 2–4 Hz dominant frequency of the state (3) is denoted by “SWD” in (**B**). (**C**) A specific bifurcation process of *ϕ*_*s*_ by setting −*ν*_*sr*_ = 2.6 mV s in (**A**). (**D**) The MFRs of SRN, obtained by setting −*ν*_*sr*_ = 2.6 mV s in Fig. 5A. It is consistent with Fig. 5C.

**Figure 6:**
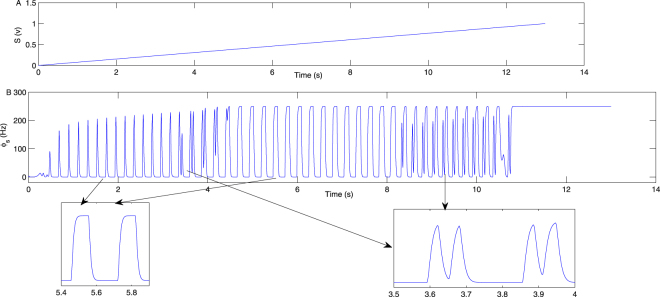
(**A**) The linear increase of V. (**B**) The time series transition process of *ϕ*_*s*_ with a linear increase in V, obtained by setting −*ν*_*sr*_ = 2.6 mV s in Fig. 5A. It is shown that the time series bifurcation diagram Fig. 6B compares very well with the state bifurcation diagram Fig. 5C.

Figure 5A,B are the state and dominant frequency analysis in the plane (−*ν*_*sr*_, *S*), respectively. S represents the external stimuli voltage applied on the SRN. It is also clear that epilepsy can be inhibited by either increasing or decreasing the value of S, as shown by the bidirectional arrows in Fig. 5A. This is a bidirectional control phenomenon as well. The dominant frequency of the seizure falls approximately in 2–4 Hz, denoted as “SWD” in Fig. 5B. Figure 5C is a typical bifurcation transition of *ϕ*_*s*_ obtained by setting −*ν*_*sr*_ = 2.6 mV s in Fig. 5A. Figure 5D presents the MFRs of the SRN, obtained by setting −*ν*_*sr*_ = 2.6 mV s in Fig. 5A. Figure 6A exhibits the linear increase of V. Figure 6B is a time series bifurcation process of *ϕ*_*s*_ with a linear increase in V, obtained by setting −*ν*_*sr*_ = 2.6 mV s in Fig. 5A. It is shown that the time series bifurcation diagram Fig. 6B compares very well with the state bifurcation diagram Fig. 5C.

S is an excitatory input to the SRN, which can induce two different effects when it is applied on the SRN. S1: it enhances the activation level of the SRN by transferring the signal along with the excitatory pathway “*SRN* → *EPN* → *SRN*”; S2: it may reduce the activation level of SRN by two inhibitory pathways “*SRN* → *TRN* → *SRN*” and “*SRN* → *EPN* → *TRN* → *SRN*” at the same time. Therefore, the bidirectional control phenomenon is exhibited by the competition mechanism between S1 and S2.

### Control of absence seizures by the external stimulation on the TRN and EPN

**Figure 7:**
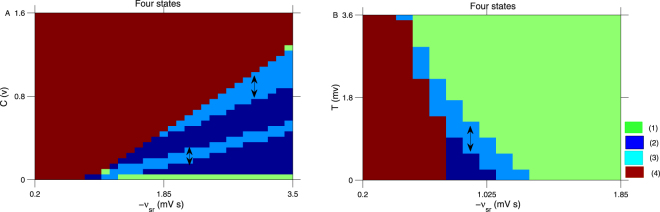
(**A**) The state transition analysis in the plane (−*ν*_*sr*_, *C*). C represents the external stimuli on the EPN. (**B**) The state transition analysis in the panel (−*ν*_*sr*_, *T*). T is the external stimuli applied on the TRN. The seizure may be inhibited by both decreasing and increasing the strength of C or T, as indicated by the bidirectional arrows in A and B.

Figure 7A demonstrates the state transition analysis in the plane (−*ν*_*sr*_, *C*). C represents the external voltage employed on the EPN. C is an excitatory input to the EPN, which may also induce two different effects. C1: it will enhance the activation level of SRN by the excitatory pathway “*EPN* → *SRN*”; C2: it may also reduce the activation level of SRN by both inhibitory pathways “*EPN* → *SRN* → *TRN* → *SRN*” and “*EPN* → *TRN* → *SRN*”. Therefore, the bidirectional control phenomenon is also observed in Fig. 7A by the competition mechanism between C1 and C2.

Figure 7B is the state bifurcation diagram in the panel (−*ν*_*sr*_, *T*). T is the external stimulus voltage employed on TRN. Because T is an excitatory stimulus to the TRN, it can reduce the activation level of SRN. Therefore, SRN enters into the low frequency firing when T is sufficiently large. However, when −*ν*_*sr*_ is relatively small (less than approximately 1 mV s, as shown in Fig. 7B), the SRN may be in the saturated state or the simple shock state. Therefore, in this case, the bidirectional control effect appears by tuning T, as indicated by the bidirectional arrow in Fig. 7B.

## The effect of the delay *τ* on absence seizures

**Figure 8:**
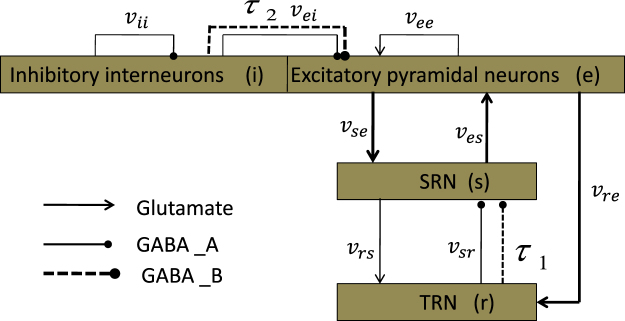
The framework structure of the model modified from Fig. 1. *τ*_1_ and *τ*_2_ are two delay parameters on the pathway “*TRN* → *SRN*” and “*IIN* → *EPN*”, respectively.

The transmission of signals often has hysteresis effects, especially in inhibitory pathways. As shown in many previous studies involving the epileptic seizure appearing on the EPN^13,15,20,37–43^, after the *GABA*_*A*_ receptor-induced inhibition begins to inhibit the SRN neurons by the inhibitory pathway “*TRN* → *SRN*”, we know that they require some time to recovery their firing rates to the rising state in each oscillation period of the SRN. Thus, the next spiking peak can be produced on the SRN when the delay *τ* is longer than this restore time, which caused by the *GABA*_*B*_ receptor-induced inhibition. Therefore, the model needs a sufficiently long *τ* to guarantee that the generation of SWDs; on the other hand, a longer delay may result in relatively more active firing of the EPN in the second messenger process. In this section, we introduce the delay parameter in the pathways “*IIN* → *EPN*” and “*TRN* → *SRN*” to study the effect of *τ* on absence seizures observed on the SRN. The model used in this section is shown in Fig. 8, and the revised equations are presented as follows,

15 $$\frac{d{\varphi }_{e}(t)}{dt}={\dot{\varphi }}_{e}(t)$$

16 $$\frac{d{\dot{\varphi }}_{e}(t)}{dt}={\gamma }_{e}^{2}[-{\varphi }_{e}(t)+F({V}_{e}(t))]-2{\gamma }_{e}{\dot{\varphi }}_{e}(t)$$

17 $$\frac{d{\varphi }_{s}(t)}{dt}={\dot{\varphi }}_{s}(t)$$

18 $$\frac{d{\dot{\varphi }}_{s}(t)}{dt}={\gamma }_{s}^{2}[-{\varphi }_{s}(t)+F({V}_{s}(t))]-2{\gamma }_{s}{\dot{\varphi }}_{s}(t)$$

19 $$\frac{dX(t)}{dt}=\dot{X}(t),\,X(t)={[{V}_{e}(t),{V}_{r}(t),{V}_{s}(t)]}^{T}$$

20 $$\frac{d{\dot{V}}_{e}(t)}{dt}=\alpha \beta ({v}_{ee}{\varphi }_{e}+{v}_{ei}F({V}_{e})+{v}_{ei}F({V}_{e}(t-{\tau }_{2}))+{v}_{es}{\varphi }_{s}(t)-{V}_{e}(t))-(\alpha +\beta ){\dot{V}}_{e}(t)$$

21 $$\frac{d{\dot{V}}_{r}(t)}{dt}=\alpha \beta ({v}_{re}{\varphi }_{e}+{v}_{rs}{\varphi }_{s}(t)-{V}_{r}(t))-(\alpha +\beta ){\dot{V}}_{r}(t)$$

22 $$\frac{d{\dot{V}}_{s}(t)}{dt}=\alpha \beta ({v}_{se}{\varphi }_{e}+{v}_{sr}^{A}F({V}_{r})+{v}_{sr}^{B}F({V}_{r}(t-{\tau }_{1}))-{V}_{s}(t))-(\alpha +\beta ){\dot{V}}_{s}(t)$$

**Figure 9:**
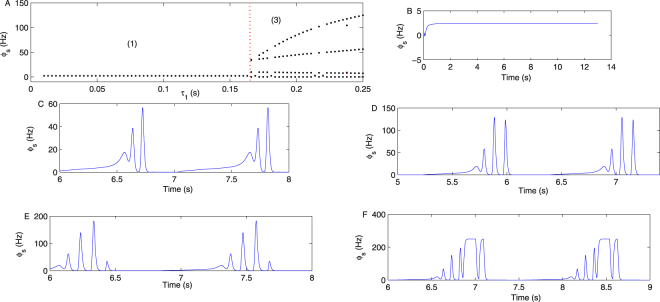
(**A**) The bifurcation diagram of *ϕ*_*s*_ by varying the parameter *τ*_1_. *τ*_1_ represents the information transfer delay from the TRN to the SRN, induced by the *GABA*_*B*_ receptor. The state (3) appeared by tuning the *τ*_1_ to approximately 0.16 s. (**B**–**F**) Five specific time sequences of *ϕ*_*s*_, obtained by setting *τ*_1_ = 0.07 s, *τ*_1_ = 0.18 s, *τ*_1_ = 0.26 s, *τ*_1_ = 0.29 s and *τ*_1_ = 0.35 s, respectively. We observe that the poly-spike wave appears when *τ*_1_ is sufficiently large, such as in Fig. 9D–F. In all simulations, we set −*ν*_*sr*_ = 0.95 mV s, *ν*_*se*_ = 1.5 mV s, *γ*_*s*_ = 160 Hz, and *τ*_2_ = 0 s.

Figure 9A is the bifurcation diagram of *ϕ*_*s*_ by varying the parameter *τ*_1_ on the pathway “*TRN* → *SRN*”. When the *τ*_1_ is larger than 0.16 s, the typical SWDs will appear, such as in Fig. 9C, obtained by setting *τ*_1_ = 0.18 s. When *τ*_1_ is sufficiently large, the poly-spike waves will appear on the SRN. For example, Fig. 9D is a three-poly-spike wave time sequence diagram obtained by setting *τ*_1_ = 0.26 s. Figure 9E is a four-poly-spike wave time sequence diagram obtained by setting *τ*_1_ = 0.29 s. Figure 9F is obtained by setting *τ*_1_ = 0.35 s.

**Figure 10:**
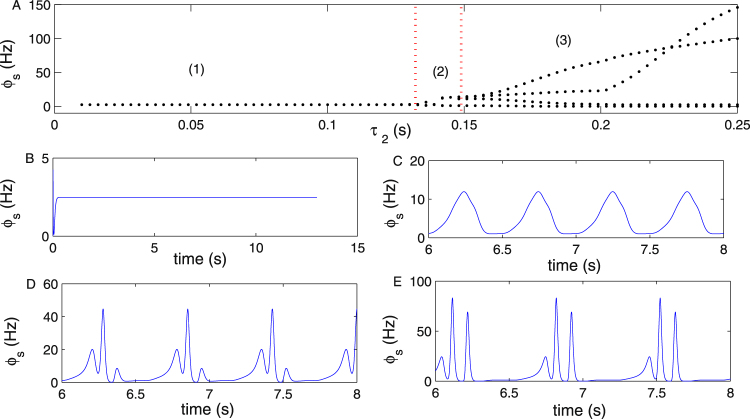
(**A**) The state bifurcation diagram of *ϕ*_*s*_ obtained by changing the parameter *τ*_2_. *τ*_2_ is the information transfer delay from inhibitory interneurons IIN to the EPN, which may be induced by the *GABA*_*B*_ receptor in IIN. The SRN enters the seizure state when *τ*_2_ is increased to approximately 0.15 s. (**B**–**E**) Four specific time sequence diagrams of *ϕ*_*s*_ are obtained by setting *τ*_2_ = 0.02 s, *τ*_2_ = 0.14 s, *τ*_2_ = 0.18 s and *τ*_2_ = 0.22 s, respectively. In all simulations, we set −*ν*_*sr*_ = 0.95 mV s, *ν*_*se*_ = 1.5 mV s, *γ*_*s*_ = 160 Hz, and *τ*_1_ = 0 s.

Figure 10A is the state transition of *ϕ*_*s*_ by changing the parameter *τ*_2_ on the pathway “*IIN* → *EPN*”. With increasing *τ*_2_, three states (1), (2) and (3) appear on the SRN. The typical SWDs emerge only when *τ*_2_ is larger than 0.16 s. Figure 10B–E are four specific time sequence diagrams of *ϕ*_*s*_, obtained by setting *τ*_2_ = 0.02 s, *τ*_2_ = 0.14 s, *τ*_2_ = 0.18 s and *τ*_2_ = 0.22 s, respectively.

**Figure 11:**
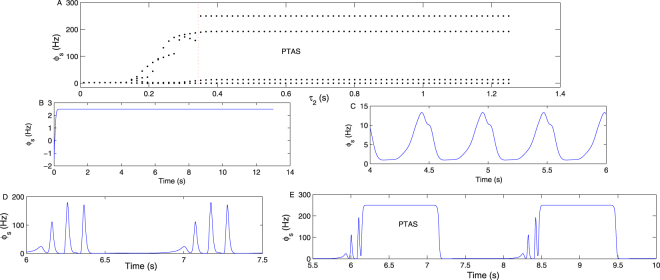
(**A**) The bifurcation transition of *ϕ*_*s*_ by increasing the parameter *τ*_2_. *τ*_2_ represents the information transfer delay from inhibitory interneurons IIN to the EPN, which may be caused by the *GABA*_*B*_ receptor in IIN. (**B**–**E**) Four typical time sequence diagrams of *ϕ*_*s*_, obtained by setting *τ*_2_ = 0.024 s, *τ*_2_ = 0.145 s, *τ*_2_ = 0.3 s and *τ*_2_ = 0.8 s, respectively. It is shown that the poly-spike wave is produced only when *τ*_2_ is sufficiently large, such as in Fig. 11D,E. We set −*ν*_*sr*_ = 0.95 mV s, *ν*_*se*_ = 1.5 mV s, *γ*_*s*_ = 160 Hz, and *τ*_1_ = 0 s in all simulations.

Figure 11A is the state bifurcation diagram of *ϕ*_*s*_ obtained by varying the parameter *τ*_2_ on the pathway “*IIN* → *EPN*” with relatively large delays. Figure 11B–E are four specific time sequence diagrams of *ϕ*_*s*_ obtained by setting *τ*_2_ = 0.024 s, *τ*_2_ = 0.145 s, *τ*_2_ = 0.3 s and *τ*_2_ = 0.8 s, respectively. We can see that the poly-spike wave appears when *τ*_2_ is sufficiently large, such as in Fig. 11D, which is a three-poly-spike wave. The SRN shows the period transfer process between the typical absence seizure state and the saturated shock state (PTAS) when *τ*_2_ is further increased, such as in Fig. 11E.

## Conclusion

**Figure 12:**
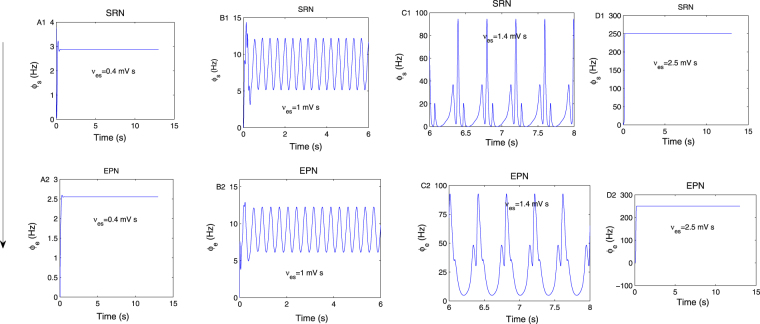
The synchronous resonance behaviours of SRN and EPN populations, which were obtained by setting *ν*_*es*_ = 0.4 mV s, *ν*_*es*_ = 1 mV s, *ν*_*es*_ = 1.4 mV s and *ν*_*es*_ = 2.5 mV s, respectively. They show that the amplitude and frequency of firing activities may be reduced when transferred from the SRN to the EPN.

In this paper, we used a model modified from mean-field network composed of the thalamus and cortex to study the mechanism of the absence seizures appearing on the SRN. In this model, the nonspecific subthalamic input^13,15,20^ onto SRN is omitted, and this does not affect the qualitative dynamical behavior of populations. The axons of SRN neurons are set to be sufficiently long to produce obvious spike waves on SRN, which has not been considered in previous studies. Through numerical calculations, we find that the typical SWDs appear on SRN by tuning all of coupling strengths of the thalamocortical network and the delay on the two inhibitory pathways. The onset mechanism can be well explained from model itself, and we have described it in details in Sections 3–5. Though these similar phenomena also appeared in some previous studies, such as^20^, they all considered the seizure appearing in the cortex. But they may have different onset mechanisms, which are studied in this paper. For example, the pathway “*TRN* → *SRN*” can induce seizures both in our model and in^20^, but it affects the activity of SRN directly, and affects the activity of cortex indirectly through the pathway “*TRN* → *SRN* → *EPN*”, which may represent different mechanisms. On the other hand, the change of synaptic connection strengths will affect the firing activities of neurons through the coupling strength between neural populations, or the activation level of different neural populations. Therefore, the seizure appearing in the cortex may be generated independently, or spread from the SRN. And, in our simulations, we find that the cortex and thalamus population could appear to have synchronous resonance activities, such as Fig. 12, which also indicate that the amplitude and frequency of firing activities may be reduced when transferred from SRN to EPN. Thus, sometimes, the cortex and thalamus may form a resonance system to affect the activity of epilepsy, which supports previous theoretical results. These seizures all can be controlled by the external stimulation acted on EPN, SRN and TRN, and the bidirectional control phenomenon is obvious. The control mechanisms are interesting and are different from the previous model studies^12–20,37–43^, which have been discussed details in Section 4. It should also be pointed out that *ν*_*es*_ and −*ν*_*sr*_ are selected as two representative pathways to explore the control mechanism. Moreover, our method can also be extended to control seizures induced by other coupling strengths and delays.

The delay is the critical factor for producing a poly-spike wave. As reported in many previous studies involving the epileptic seizure appearing on the EPN, such as^20^, and as analyzed in the section of 5, in the second messenger process, the number of pulse extremum points will increase in one period with a longer delay, such as in Fig. 4(a) in^20^. However, we observed that the minimum delay to produce seizures is larger in the current study (>0.16 s) than in^20^ (>0.04 s). As is well known, the output of the TRN affects the activation level of the SRN through the direct inhibitory pathway “*TRN* → *SRN*”; however, it affects the stability of the EPN through the inhibitory pathway “*TRN* → *SRN*” and the excitatory pathway “*SRN* → *EPN*”. The signal transmitted from the TRN to the EPN requires a relatively longer time than that from the TRN directly to the SRN. Thus, the delay is required to generate seizures, and it is longer in this paper than in^20^. Moreover, in this paper, we observed additional complex spike-wave phenomena, such as four-poly-spike-waves. We think that they may be more prone to appear on the SRN than on the EPN; otherwise, when the seizure activity is transferred from the SRN to EPN, the number of poles would decrease. The *GABA*_*B*_ receptor pathways have been reported as the basic mechanisms mediating epilepsy seizures in many studies^44,45^. However, after careful investigation, we find that there are few studies on *GABA*_*B*_ in the cortex inducing absence seizure; thus, we hope that the results obtained in Figs 10, 11 and 12 can inspire further experimental studies.

The source of data employed in simulations has been denoted in appendix. They are all taken in the reasonable biology range, which can also be found in previous studies. For example, the physiologically allowed ranges for ${Q}_{e}^{max}$, ${Q}_{i}^{max}$, ${Q}_{s}^{max}$ and ${Q}_{r}^{max}$ are 100–1000 Hz^12^, and in this model, they are standardized as 250 Hz. The physiological values of *θ*_*e*_, *θ*_*i*_, *θ*_*s*_ and *θ*_*r*_ are about 15 mV^12^. Generally, the rational biology range of *γ*_*e*_ is 70–150 Hz^12^, and we take it as 100 Hz in our simulations. *γ*_*s*_ is estimated to be 150 Hz in the model; however, it was not thoroughly evaluated in previous studies. We think this value is reasonable, as referred to the range of *γ*_*e*_. The time delays are varied by changing the brain environment; unless otherwise noted, we take *τ* = 0.07 s in simulations, which is appropriate as described in^20^. *α*, *β* and *σ* are usually set as constants, and the values used in the paper also appeared in previous studies when considering absence epilepsy, such as in^43^. The reasonable biological range of the coupling strengths *ν*_*ee*_, *ν*_*ei*_, *ν*_*re*_, *ν*_*rs*_, *ν*_*sr*_, *ν*_*es*_ and *ν*_*se*_ is 0.05–10 mV s^12^, and hence, we think that these values were reasonably used in this new model.

In^46^, Hu *et al*. first explored the control mechanism of absence epilepsy appearing in the SRN in a corticothalamic-basal ganglia network in which the seizure was induced by the inhibitory pathway “*TRN* → *SRN*” and inhibited by the basal ganglia. However, as described in the introduction, absence epilepsy seizures are caused by abnormal interactions in the CT network, and accurate onset mechanisms are still unclear. Therefore, in this paper, we first systematically explore the onset mechanism of absence epilepsy seizures appearing in the SRN in a modified CT mean-field network. We find that absence seizures can also be induced by other excitatory and inhibitory pathways in the CT network, which may represent different mechanisms and which have been explained in detail in the paper. It is noted that the effect of delay in the pathway “*IIN* → *EPN*” on seizures has not been involved in previous model studies, the mechanisms of which have been explored in depth in our CT model. Furthermore, we also study the control effect by directly employing the external stimulus voltage on the TRN, SRN and EPN, the mechanism of which has not been discussed in the previous study. Therefore, in this paper, we may provide a unified theoretical framework to study the mechanism of absence seizures appearing in the SRN in the CT model in the future.

**Figure 13:**
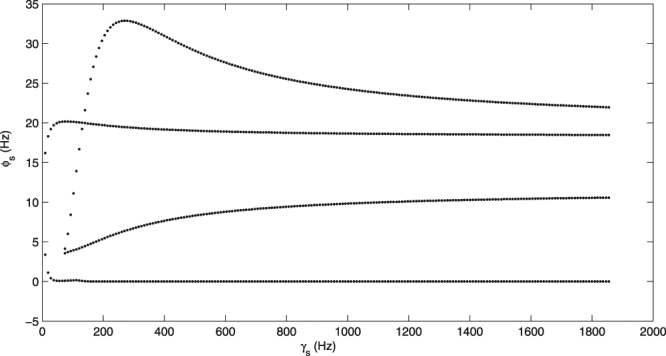
The transition process of *ϕ*_*s*_ as the increase of *γ*_*s*_. *γ*_*s*_ is the damping rate of SRN. We set −*ν*_*sr*_ = 1.2 mV s in the simulation.

It should be noted that the mean-field model is an idealized mathematical model, generally used to facilitate the numerical calculation and to study the qualitative properties of neuron firing activity. The brain environment is very complicated, and several other factors, such as, the synaptic plasticity, the connection structure between neurons, and the delay on excitatory pathways, may all have great influences on seizures in the brain. For example, in our model, we find that the damping rate of the SRN has little effect on seizures (Fig. 13); however, as is well known, the damping rates of different neurons are different and may have important effects on neuronal activity. These studies can only be resorted to the spiking neural network model in the future. Another important question is the control of epileptic seizures. There are many theoretical research results on the control mechanism of epileptic seizures appearing on the cortex by the basal ganglia^37–42^. Although, we have observed some qualitative control mechanisms in our model by employing external stimuli, more refined studies through the internal regulation of the brain need to be conducted in our future studies.

Finally, the main work of the paper is to provide the generation and inhibition mechanisms of absence epilepsy appearing on the SRN, and our model and methods can also be applied to study other types of seizures, such as spasmodic attacks, secondary generalized seizures and juvenile myoclonic epilepsy. They might also be generated on the SRN and have been rarely studied in previous literatures. The transfer between different types of epileptic seizures may also be an interesting research topic in the future. In light of all the parameters used in the numerical calculation taken from real experimental data, the result obtained in this paper might help us to better understand the mechanism of absence epilepsy appearing in the CT network. On the other hand, in addition to the model-driven approaches described in this paper, data-driven approaches, such as network biomarkers^47–53^ and dynamic network biomarkers^54–56^ based on the high throughput omics data, can also be applied to this field.

## Electronic supplementary material

Supplementary Information
