# Supplementary material for: The generation mechanism of spike-and-slow wave discharges appearing on thalamic relay nuclei
Source: Sci Rep. 2018 Mar 21;8:4953. doi: 10.1038/s41598-018-23280-y (PMC5862852; doi:10.1038/s41598-018-23280-y)
Supplement: Supplementary file 1 — Supplementary Information [file 41598_2018_23280_MOESM1_ESM.pdf]

The generation mechanism of spike-and-slow wave discharges appearing on thalamic relay nuclei

Bing Hu[1,2*], Yu Guo[1], Feng Shi[1], Xiaoqiang Zou[1], Jing Dong[1], Long Pan[1], Min Yu[1], Chaowei Zhou[1], Zhang Cheng[1], Wanyue Tang[1], Haochen Sun[1], Luonan Chen[2*]

[1] *Institute of Applied Mathematics, Department of Mathematics and Statistics, College of Science, Huazhong Agricultural University, Wuhan 430070, China;*

[2] *Key Laboratory of Systems Biology, CAS center for Excellence in Molecular Cell Science, Innovation Center for Cell Signaling Network, Institute of Biochemistry and Cell Biology, Shanghai Institute of Biological Sciences, Chinese Academy of Sciences, Shanghai 200031, China*

1. Appendix

Unless otherwise noted, we employed the follow parameter values for numerical computations,

Parameter	Meaning of parameter	Parameter values	Sources
Q_e^{max}, Q_i^{max}	Cortical maximum firing rate	250 Hz	[12,20,37,43]
Q_s^{max}	SRN maximum firing rate	250 Hz	[12,20,37,43]
Q_r^{max}	TRN maximum firing rate	250 Hz	[12,20,37,43]
θ_e, θ_i	Mean firing threshold of cortical populations	15 mV	[12,20,37,43,53]
θ_s	Mean firing threshold of SRN	15 mV	[12,20,37,43,53]
θ_r	Mean firing threshold of TRN	15 mV	[12,20,37,43,53]
γ_e	Cortical damping rate	100 Hz	[12,20,37,43]
γ_s	SRN damping rate	150 Hz	Estimated
τ	Time delay due to slow synaptic kinetics of $GABA_B$	70 ms	[20]
α	Synaptodendritic decay time constant	50 s^{-1}	[12,20,37,43,53]
β	Synaptodendritic rise time constant	200 s^{-1}	[12,20,37,43,53]
σ	Threshold variability of firing rate	6 mV	[12,37,43,53]

Coupling strength	The output nuclei	The receiving nuclei	Parameter values	Sources
ν_{ee}	EPN	EPN	1.1 mV s	[12,20,43]
ν_{ei}	IIN	EPN	-1.6 mV s	[12,20,43]
ν_{re}	EPN	TRN	0.05 mV s	[12,20,37]
ν_{rs}	SRN	TRN	0.55 mV s	[12,20]
$\nu_{sr}^{A,B}$	TRN	SRN	-0.55 mV s	[12,53]
ν_{es}	STN	EPN	2 mV s	[12,20]
ν_{se}	EPN	SRN	2 mV s	[12]
